# Correction: A mathematical model describing the localization and spread of influenza A virus infection within the human respiratory tract

**DOI:** 10.1371/journal.pcbi.1008424

**Published:** 2020-11-02

**Authors:** Christian Quirouette, Nada P. Younis, Micaela B. Reddy, Catherine A. A. Beauchemin

In Fig 3 there is an error in the text in the legend within the figure for parts d, e, f. It says 1 dpi, 2 dpi, 3 dpi, 4 dpi, 7 dpi, 7 dpi, but it should say 0 dpi, 1 dpi, 2 dpi, 3 dpi, 4 dpi, 7 dpi. The authors have provided a corrected version here.

**Fig 3:**
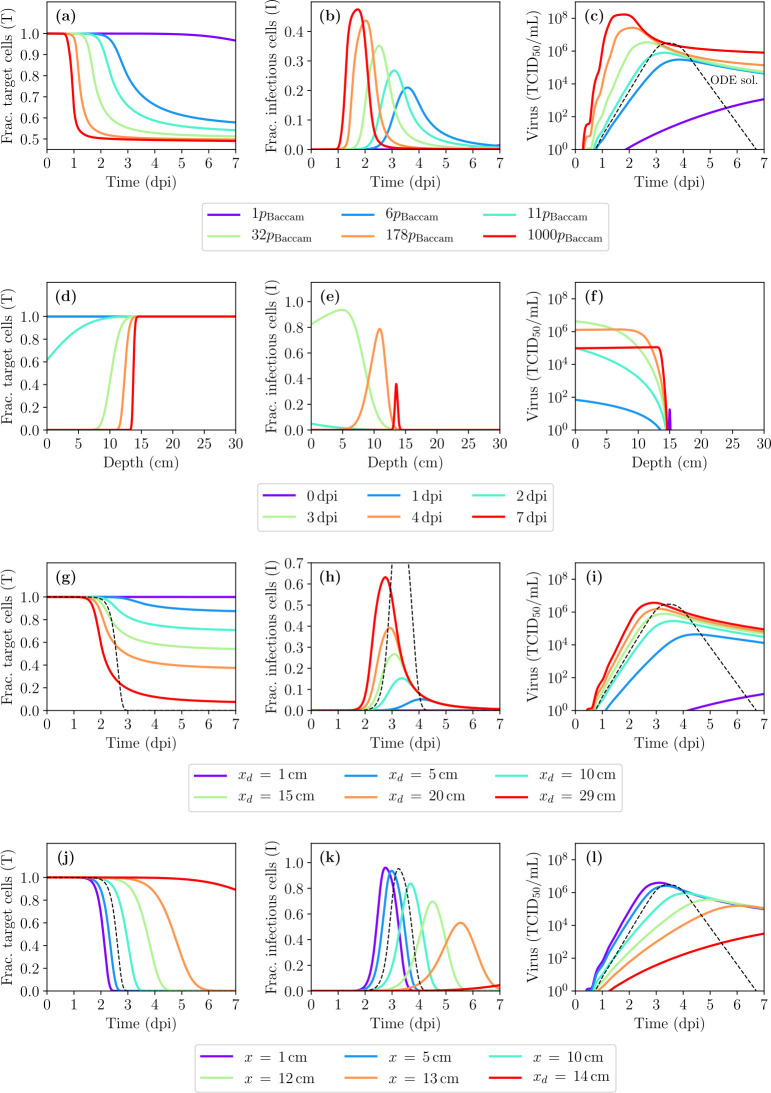
IAV infection kinetics in the presence of diffusion and advection. (a,b,c) Time course (averaged over space) of the infection for the fraction of cells in the (a) target/uninfected or (b) infectious state, and (c) the infectious virus concentration, obtained using the ODE MM (dashed) or the spatial MM (solid), as the rate of virus production, *p*, is varied. (d,e,f) Localized fraction of cells in the (d) target or (e) infectious state and (f) the infectious virus concentration as a function of depth within the HRT shown at specific days post-infection (dpi). (g,h,i) Same as (a,b,c) but varying the depth of deposition of the initial virus inoculum (*x*_*d*_). (j,k,l) Similar to (a,b,c) but rather than being averaged over space, the infection time course is shown at specific, spatially localized depths (*x*). Unless otherwise noted, *p* = 11 × *p*_Baccam_ and *x*_*d*_ = 15 cm.
