# Correction: A mathematical model describing the localization and spread of influenza A virus infection within the human respiratory tract

**DOI:** 10.1371/journal.pcbi.1009851

**Published:** 2022-02-04

**Authors:** Christian Quirouette, Nada P. Younis, Micaela B. Reddy, Catherine A. A. Beauchemin

In Fig 7 there is an error in the figure caption. The correct figure caption is provided below.

**Fig 7. The effect of cellular regeneration on the MM prediction of IAV infection kinetics**. The effect of varying (a–c) the regeneration rate (*r_D_*) or (d–f) the regeneration delay (*τ_D_*) are shown. Unless varied, *τ_D_* = 1 d and *r_D_* = 0.75 d^-1^.
