# Supplementary material for: A mathematical model describing the localization and spread of influenza A virus infection within the human respiratory tract
Source: PLoS Comput Biol. 2020 Apr 13;16(4):e1007705. doi: 10.1371/journal.pcbi.1007705 (PMC7179943; doi:10.1371/journal.pcbi.1007705)
Supplement: S1 Fig — The effect of the length, measured from x = 0 down to x = Ltop, over which V(x, t) in the spatial MM is averaged to produce the curves showing Virus versus Time, for variants of the MM (a) without cellular regeneration and a full immune response; (b) with cellular regeneration but without a full immune response; and (c) with cellular regeneration and a full immune response. (PDF) [file pcbi.1007705.s004.pdf]

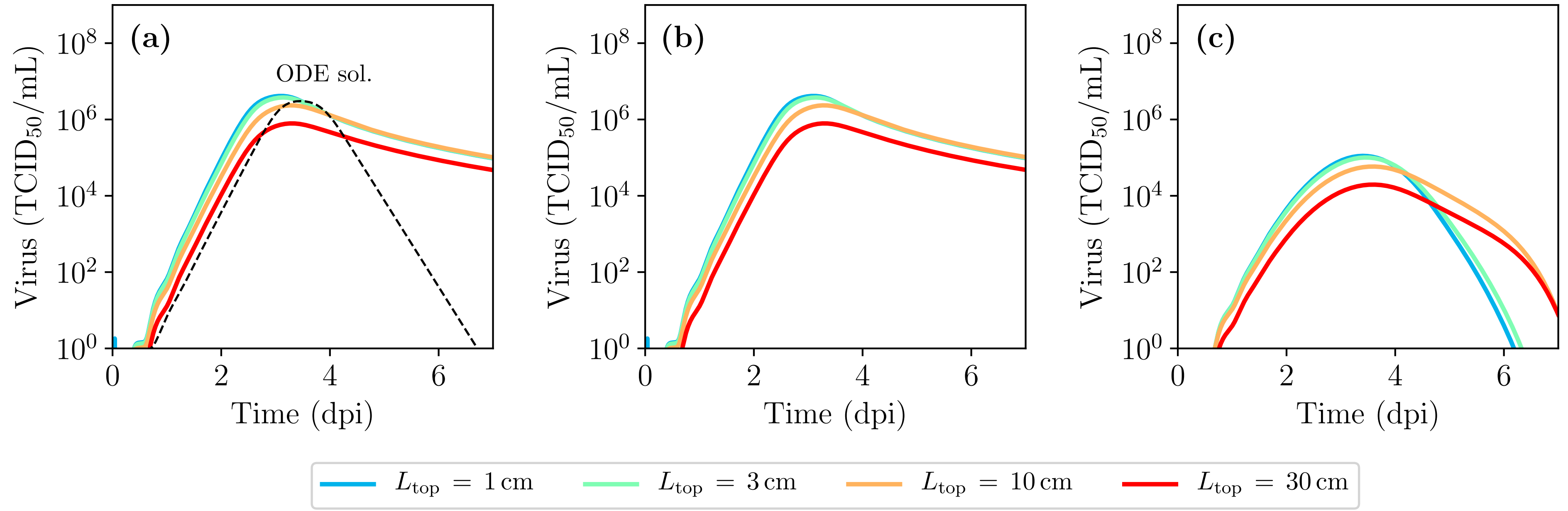

S1 Figure. Effect of varying the length over which the virus concentration is measured. The effect of the length, measured from $x = 0$ down to $x = L_{\mathrm{top}}$, over which $V(x,t)$ in the spatial MM is averaged to produce the curves showing Virus versus Time, for variants of the MM (a) without cellular regeneration and a full immune response; (b) with cellular regeneration but without a full immune response; and (c) with cellular regeneration and a full immune response.
