# Supplementary material for: A mathematical model describing the localization and spread of influenza A virus infection within the human respiratory tract
Source: PLoS Comput Biol. 2020 Apr 13;16(4):e1007705. doi: 10.1371/journal.pcbi.1007705 (PMC7179943; doi:10.1371/journal.pcbi.1007705)
Supplement: S2 Fig — Whereas Fig 3(g)–3(i) shows the effect of deposition depth in the earliest version of the MM with diffusion and advection alone, these graphs show the effect of deposition depth of the initial virus inoculum (xd) in the complete spatial MM which includes cellular regeneration and a full immune response. (PDF) [file pcbi.1007705.s005.pdf]

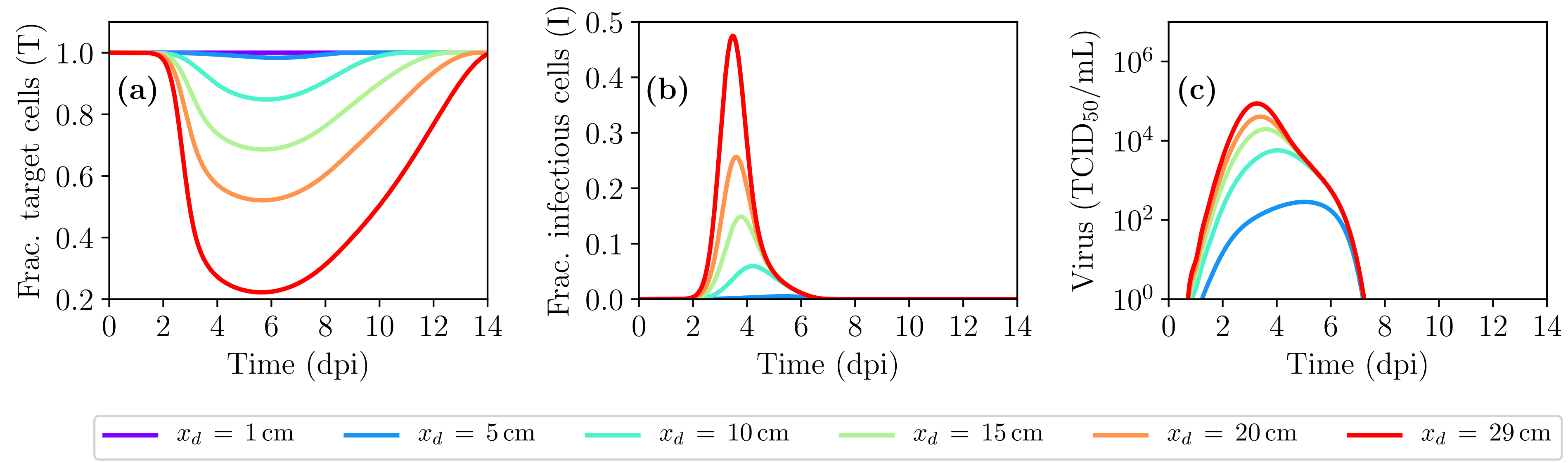


**S2 Figure. Effect of the inoculum deposition depth on the complete spatial MM.** Whereas Fig 3(g–i) shows the effect of deposition depth in the earliest version of the MM with diffusion and advection alone, these graphs show the effect of deposition depth of the initial virus inoculum ($x_d$) in the complete spatial MM which includes cellular regeneration and a full immune response.
