# Supplementary material for: A mathematical model describing the localization and spread of influenza A virus infection within the human respiratory tract
Source: PLoS Comput Biol. 2020 Apr 13;16(4):e1007705. doi: 10.1371/journal.pcbi.1007705 (PMC7179943; doi:10.1371/journal.pcbi.1007705)
Supplement: Attachment — Submitted filename: review-response.pdf [file pcbi.1007705.s006.pdf]

Response to reviewers for PCOMPBIOL-D-19-01437

A mathematical model describing the localization and spread of influenza A virus infection within the human respiratory tract

Christian Quirouette, Nada P. Younis, Micaela B. Reddy, Catherine A.A. Beauchemin

December 10, 2019

We would like to thank the reviewers for their helpful comments which enabled us improve our manuscript. Below, we respond to the reviewers' comments, point-by-point, indicating where in the manuscript corrections were made to address the comments, where applicable.

Associate Editor

Comment 1. I enjoyed reading the paper. The work certainly tackles an important problem – the effect of spatial considerations on the dynamics of infection. I like the way the paper has addressed an important aspect of this problem. There is one aspect that I believe could be expanded on – the assumptions of the model. Reviewer 1 has I think gone into this in detail and I think addressing these would substantially facilitate others in building on your very nice paper.

Thank you. We believe we have addressed all reviewers' concerns in details below, and especially with regards to key assumptions of our model.

Reviewer #1

Comment 2. One assumption I found puzzling is that the infection starts at 15cm (in the middle) of the HRT. Is it consistent with the initial infection site of a typical influenza infection? The authors showed that when the position of this initial infection site is higher (x is smaller than 15cm), the model predicts only a very small fraction of target cells are infected due to the high advection rate (Fig. 3 j-l and Fig. 4). I think this is a prediction inconsistent with an actual influenza infection, where the initial infection site is higher in the HRT than assumed in the model, and the establishment of infection shall not be very sensitive to where it starts. I think it is important to use a more biologically plausible value for where the infection starts at the HRT in simulations.

To our knowledge, the "typical" site in the HRT where influenza A virus infection is initiated has not been identified. We now comment on this issue in more details in the Discussion in a manner that we believe specifically addresses the concerns raised by the reviewer and the potential impact of this choice on our results:

> *"Herein, the depth of initial inoculum deposition in the MM was set arbitrarily to* 15 cm*. In reality, the most likely site of deposition would be very difficult to determine. There are many possible routes for IAV transmission — e.g. inhalation of an expulsion spray from a sneeze or a cough, direct contact with a contaminated person, or by indirect contact with a contaminated object — all of which are believed to play some role, with no consensus on the most likely or most common route [16]. Even if the route of transmission is known or assumed, for example from a cough, there is a large range of potential particle sizes for this mode of transmission [22], and particle size is believed to be inversely proportional to depth of deposition [7]. Even if the most likely depth of deposition was known, the site of initial particle*

deposition is unlikely to be the same as the site at which infection will first take hold because virus particles that deposit along the airways have to traverse the mucus and PCF layers to reach infection-susceptible epithelial cells, and advection in those layers would quickly drag virus away from its initial deposition site."

The corresponding caption for the S2 Figure, added as Supplementary Information, is as follows:

> " **S2 Figure. Effect of the inoculum deposition depth on the complete spatial MM.** *Whereas Fig 3(g–i) shows the effect of deposition depth in the earliest version of the MM with diffusion and advection alone, these graphs show the effect of deposition depth of the initial virus inoculum (x_d) in the complete spatial MM which includes cellular regeneration and a full immune response."*

Comment 3. My interpretation of the inconsistency above is that other parameters used in the model may be unrealistic. For example, the advection rate in the work (40 mm/s) is taken from estimates of the advection rate for periciliary liquid (ref. 47 within the manuscript). However, it is highly likely that the viral particles get tracked in the tissue while they travel with the periciliary liquid, leading to a smaller advection rate for viruses. It is not unreasonable to speculate that the advection rate for viruses would be an order of magnitude smaller than the periciliary liquid due to the complicated structure of the epithelium cells on the surface of HRT. Therefore, given the uncertainty in the advection rate, it is crucial to evaluate the robustness of all the results against variations in the advection rate.

The advection speed measurements from what was previously ref. 47 were not direct measurements of the periciliary liquid itself, but rather the speed of microspheres 0.2 μm in diameter within the PCF, which should be akin to the speed experienced by free (not cell attached) IAV particles 0.1 μm in diameter within the PCF. So we do believe 40 μm/s is a biologically realistic and appropriate estimate for IAV advection speeds in the PCF in vivo. This being said, the reviewer makes an excellent point in suggesting that we should evaluate the impact of this parameter on our findings. We have added the following paragraph to the end of the Results section on IAV kinetics in the presence of virus diffusion and advection:

> ***"Finally, the robustness of these results to variations in the advection speed was evaluated. Fig 5(a–c) shows the effect of the advection speed on the infection kinetics when the virus production rate is fixed to its value in the non-spatial model,*** p_{Baccam}***. In Fig 5(d–f), the virus production rate is increased as advection speed is increased so that the spatial MM approximately reproduces the timing of viral titer peak in the non-spatial MM for all values of the advection speed. For the lowest advection speed considered (***$v_a = 4$ μm/s***), no adjustment to the virus production rate was needed. Fig 5(g–i) shows the spatial extent of the infection at specific days post-infection for advection speed*** $v_a = 4$ μm/s ***and virus production rate*** p_{Baccam}***. Even at a speed*** $10\times$ ***slower than that measured experimentally [18], the MM predicts advection would still prevent the infection from travelling beyond its initial deposition depth (herein*** $x_d = 15$ cm***), and the infection still appears to be progressing downwards as the infection peaks and resolves faster in the upper than in the lower HRT."***

In light of this, given that the virus production rate required to counter the effect of advection in the spatial MM is dependent on the magnitude of the advection speed, and given that this value might not be well estimated, we have rephrased the abstract to read:

> ***"Diffusion was found to be negligible in the presence of advection which effectively sweeps away IAV, preventing infection from disseminating below the depth at which virus first deposits. Higher virus production rates (***10***-fold) are required at higher advection speeds (***40 μm/s***) to maintain equivalent infection severity and timing."***

Comment 4. The authors modeled the impact of the interferon response as a reduction in the viral production rate (p). However, interferon response also protects susceptible cells from infection through paracrine signaling. This can have a strong impact on the spread of viruses especially the spread is spatial (Huang, et al. Frontiers in Immunology 2019; 10, 1736; Domingo-Calap et al. Nat Microbiol. (2019) 4:1006–13.). Therefore, the conclusions about the role of the interferon response during spatial spread in this work are likely to be incorrect/incomplete. I understand extending the model explicitly with an interferon component may go beyond the scope of the study. In that case, I think the authors shall either avoid modeling the interferon response or thoroughly discuss the limitations of current formulation in the context of the existing literature.

While it is true that IFN can protect susceptible cells against infection, it is also true that it can down-regulate virus production in infected cells and recruit additional immune factors and cells to locally enhance the immune response (higher cell killing rate, higher virus neutralization). Since not all roles can be captured by a reasonably parsimonious MM, especially in the absence of extensive data from which to estimate the rates controlling these mechanisms, it is unclear which one most significantly shapes infection kinetics and thus most critically should be represented.

Herein we chose to represent the role of IFN as down-regulating virus production because it is simplest to represent mathematically. Representing the role of IFN as conferring resistance to infection poses a number of problems because it requires: (1) that IFN production be local/localized to each infected cell rather than at equal concentration everywhere as assumed herein; (2) that its diffusion, advection, production, and clearance rate (requiring one more equation and 4 more unknown parameters) be modelled along with that of the virus, but given its smaller size (molecule rather than particle), capturing the rapid rate of IFN diffusion would be extremely computationally costly; (3) knowing the local concentration of IFN, we would still need to assume/explore the rate at which its uptake by cells translates to resistance (time delay), the degree of resistance conferred, and how it maps to IFN concentration, and the rate at which this resistance wanes, i.e. at least 4 more unknown parameters. Thus, it would be non-trivial, and possibly also not meaningful given the number of unknown parameters, to include or investigate this additional role for IFN.

This being said, given this specifically spatial role for IFN (locally prevent infection), its exploration within an explicitly spatial MM would indeed be very interesting. But given the challenges we mention above, it would merit a paper on its own. Thus, to address this concern raised by the reviewer, we made the following changes. In Results, when we first discuss the role of IFN we chose to implement in our MM, we make it clearer that other roles for IFN could have been represented by now explicitly listing them:

> ***"IFN is known to have many effects [9, 13, 15], including making cells resistant to infection and recruiting additional immune factors and cells to locally enhance cell killing and virus neutralization. For simplicity, its effect herein is to reduce the rate of virus production p [1, 11], via resistance parameter, f_{50}, analogous to the IC$_{50}$ used to describe antiviral resistance."***

Later, in our Discussion, we return to this point in the context of possible future work:

> *" In the future, the addition of an explicit, dynamical immune response — to replace the empirical equations used herein as stand-ins for immune response components — would enable the use of this MM to reproduce the kinetics of re-infection, similar to the work done by others [6, 21].* ***Of particular interest would be to explore the role of IFN secreted by infected cells in inducing an antiviral state in neighbouring cells [9, 13]. This spatial MM would be ideal to explore the effect of diffusion and advection in either enhancing this spatially local role of IFN by spreading it further afield, or decreasing its role by either disseminating its concentration over a larger area or perhaps by allowing IAV to outrun IFN's localized antiviral effect. This has been explored to some extent by others in the presence of diffusion alone [9, 13], but it would be particularly interesting to revisit this process in the presence of both diffusion and upwards advection."***

Comment 5. Maybe this is not intended by the authors; but the current manuscript reads like that the spatial spread of the influenza virus as modeled using the PDEs is the only mode of spread. In reality, it is likely that the virus spread both locally (with a spatial structure) and over long distances (without a spatial structure), especially when the infection becomes systematic. I think this is one of the reasons that the previous ODE models are useful in addressing many questions, despite the spatial structure in the spread. I think the work is interesting in that it addresses the spatial issue and sheds light on the spatial aspect of the infection dynamics; at the same time, it is important to fully discuss the limitations of the PDE model and acknowledge the infection process modeled is one aspect of infection. It'd be useful to have a paragraph discussing when the PDE model is or is not appropriate/needed to model the influenza infection dynamics.

As we understand it, uncomplicated, seasonal IAV infection is normally spatially localized to the upper respiratory tract, and the upper portion of the lower respiratory tract. Only in the case of infection with severe IAV strains, or in the presence of bacterial co-infection or other severity-enhancing factors, would IAV infection become systemic, i.e. be found in organs beyond the human respiratory tract, and even then it is generally believed that virus replication is inefficient or negligible outside the respiratory tract [23]. For this reason, spatially explicitly modelling IAV infection, as done herein, is more biologically accurate. But this additional accuracy is not always required, and simplifying assumptions can sometimes be appropriate depending on the goal of the MM's application. Typical MMs of IAV, which are non-spatial, essentially assume that infection is spatially uniform within the HRT, i.e. the fraction of cells infected and the concentration of virus is approximately the same everywhere in the HRT, which is not biologically correct. However, these simplified, less accurate MMs can still reproduce infection kinetics well. Indeed, we show herein that one can get more or less the same virus concentration time course, as measured experimentally, using either MMs. For example, see Figure 3(c) wherein we make the spatial MM reproduce the same virus time course as the non-spatial ODE model (dashed line) by modifying the virus production rate between the 2 MMs.

So while both spatial and non-spatial MMs can reproduce the experimentally measured virus titer time course well, they do so for different estimates of their respective parameter values, i.e. they predict different rates for the same biological processes. To the extent that parameter values are scientifically important (e.g., as the virus production rate increases, so does the likelihood of drug resistance emergence), and using different MMs will yield different estimates of parameters, there will be contexts in which using either MM will yield the same results and predictions, and others where the simplifying assumptions made by the non-spatial MMs will lead to a skewed picture of infection kinetics. Similarly, our MM herein utilizes an overly simplistic representation of the immune response, whereas other MMs have far more sophisticated and accurate representations, and this too would affect MM parameter estimates. Since our goal herein is not parameter estimation, it is not as much an issue in our context.

Ultimately, all MMs are simplifications of the real system, but it is important to explore the consequence of these simplifications and keep them in mind when analyzing or interpreting one's results. For example, when modelling IAV infection in vivo, one should test at least to see if their results are modified when taking into account the spatial aspect of an infection, and this could be done by using our spatial MM. This was not communicated in the manuscript and so the following change has been made to the Discussion:

> " *The spatial MM presented herein, despite its simplicity, offers a novel and interesting opportunity to study the spatial localization and dissemination of IAV infections within the HRT.* ***In particular, it could serve to test if results obtained by non-spatial ODE MMs are modified when taking into account spatial aspects of IAV infections.*** "

Comment 6. I found that the section on comparing seasonal and avian influenza infections and the subsequent predictions of the impact of antivirals on these infections unconvincing. The authors adjusted model parameters such that the infection dynamics are 'somewhat' consistent with experimental data (Fig. 10). However, the authors provide no evidence demonstrating that the differences in seasonal and avian influenza are indeed because of the differences in the

parameter values of the model. Thus, the predictions of the impact of antivirals on infection dynamics should be treated as hypotheses rather than conclusions.

For most if not all parameter shifts between the human and avian portraits we point to literature results where an enhancement or decrease in that process between the two strains has been reported. Thus, our shifts are not arbitrary or hypothetical per se, but their magnitude mostly is, and admittedly the process is not rigorous because sufficient, coherent information is not available on this matter. As such, what we report herein is indeed more like hypotheses than findings. We agree that this was not made sufficiently clear in the manuscript. To make this abundantly clear, we have made changes where we first introduce our approach to modelling differences between seasonal and avian IAV strains:

> *" The IAV H5N1 portraits were constructed from the MM of the seasonal portrait by shifting parameters controlling cell-virus interactions and immunity in keeping with differences believed to be responsible for that shift. These parameters are presented in Table 2.* ***This approach was chosen due to the limited data available on IAV H5N1 infection, and is simply meant to display one of the MM's possible applications. It is not intended as proof that these differences in parameter values are responsible for the differences between a infection with seasonal or H5N1 IAV strain.*** *"*

and then later, when discussing antivirals:

> *"Fig 11 explores the impact of antiviral therapy with neuraminidase inhibitors (NAIs), administered at various times post-infection, on the time courses for infection with seasonal or avian IAV strains.* ***These results should be treated as hypotheses, as the infection time courses are hypothetical, and the results should serve as further display of the MM's possible future applications."***

Comment 7. I find the length of the manuscript is too long. It'd be better if the authors try to reduce the length of the manuscript and the number of figures, and highlights the most important aspects of the results.

We agree that the manuscript is long, but we feel that length is a matter of preference. We like all our content and figures and would like to keep it all: different aspects of the work might appeal to different readers. We would rather to leave it up to individual readers to skim through parts they are less interested in. Please see also our related response to **Comment 21**.

Reviewer #2

Comment 8. While the abstract makes sense after reviewing the entire paper, it confused me on first read. The mucus escalator and advection (not commonly used terms for modeling or virology generalists) are mentioned in the first sentence prior to being formally defined. The phrase "IAV advection dominates kinetics" is vague. It would be better to say that advection rather than diffusion is necessary to explain existing quantitative data as well as empirically derived qualitative spatial features of IAV infection. The sentence regarding antiviral therapy does not point to any particular scientific conclusion. The existence of experimental data consistent with model predictions is left unmentioned.

Given that the abstract is at most 300 words, we had to weigh what to add against what we would have to remove in making revisions. Nonetheless, we feel the revisions made improved the abstract and address all issues raised by the reviewer. Note that while we now mention the MM's predictions are consistent with experimental findings, the latter are not cited because journal guidelines do not allow citations in the abstract. The revised abstract is as follows:

> *" Within the human respiratory tract (HRT), virus diffuses through the periciliary fluid (PCF) bathing the epithelium.* ***But virus also undergoes advection: as the mucus layer sitting***

atop the PCF is pushed along by the ciliated cell's beating cilia, the PCF and its virus content are also pushed along, upwards towards the nose and mouth. *While many mathematical models (MMs) have described the course of influenza A virus (IAV) infections in vivo, none have considered the impact of both diffusion and advection on the kinetics and localization of the infection. The MM herein represents the HRT as a one-dimensional track extending from the nose down towards the lower HRT, wherein stationary cells interact with IAV which moves within (diffusion) and along with (advection) the PCF.* ***Diffusion was found to be negligible in the presence of advection which effectively sweeps away IAV, preventing infection from disseminating below the depth at which virus first deposits. Higher virus production rates (10-fold) are required at higher advection speeds (40 µm/s) to maintain equivalent infection severity and timing.*** *Because virus is entrained upwards, upper parts of the HRT see more virus than lower parts. As such, infection peaks and resolves faster in the upper than in the lower HRT, making it appear as though infection progresses from the upper towards the lower HRT, as reported in mice. When the spatial MM is expanded to include cellular regeneration and an immune response,* ***it reproduces tissue damage levels reported in patients. It also captures the kinetics of seasonal and avian IAV infections, via parameter changes consistent with reported differences between these strains, enabling comparison of their treatment with antivirals.*** *This new MM offers a convenient and unique platform from which to study the localization and spread of respiratory viral infections within the HRT."*

Comment 9. While the paper generally does a great job of reviewing existing IAV modeling literature and concepts incorporated into the model (the sections on immunity and cellular regeneration are particularly useful for the reader), the introductory paragraph regarding the mucous escalator (lines 14-16) is rather sparse. Only one paper is cited (a NEJM review which focuses mostly on the biophysical and biochemical properties of mucous). I would appreciate more detail regarding the role of cilia, the overall purpose of mucous clearance, and an explanation for why viral particles are likely to have a similar advection rate to the surrounding mucous. Specifically, the data used to inform parameter values from reference 47 (Table 1, line 57) should be described in some detail as the advection rate is central to all model conclusions. Similar detailed attention should be given to describing the diffusion rate derived from ref 37.

Along with clarifying sentences in the Abstract which will also be helpful, we revised sentences in the Introduction around (previous) lines 14–16 to provide more information on the mucociliary clearance process:

> ***"IAV infection spread faces a tremendous physical, spatial barrier in the form of the thick mucus layer which lines the HRT [5, 10]. This mucus layer sits atop the PCF, which itself sits atop and bathes the epithelial cells that line the HRT. As the ciliated epithelial cells' coordinated, beating cilia push the mucus layer upwards, it also pushes the PCF and the virus it contains, upwards towards the nose and mouth, and out of the HRT [3, 14]. Virus entering via the airways above the mucus layer, or progeny virus released at the base of the PCF by infected cells, can either become trapped by the mucus acting like flypaper, or remain in the PCF and get carried along with it, upwards and out of the HRT. These effective clearing mechanisms, jointly referred to as the mucociliary escalator, are typically taken somewhat into account in non-spatial ODE MMs via a term for the exponential clearance of virions. This simplification, however, does not account for the fact that not all cells will have equal exposure to the virus: cells that are downstream of the PCF flow (higher in the HRT) will have greater exposure to virus than those upstream, located deeper within the HRT. To our knowledge, the effect of virus entrainment due to the upwards advection of the PCF on IAV infection localization and spread within the HRT has never been evaluated."***

In Results, we revised sentences around (previous) lines 57 et al. to provide details about the procedures followed in (previous) references 37 (diffusion) and 47 (advection):

> ***"The diffusion coefficient of IAV in the PCF, D_{PCF}, is estimated based on the Stokes-Einstein equation for IAV diffusing in plasma at body temperature, namely $D_{PCF} \approx 10^{-12}$ m²/s [2, 12]. The advection speed of the PCF is set to that of the mucus layer, $v_a = 40$ µm/s, based on experiments by Matsui et al. [18] wherein microspheres 0.2 µm in diameter, located within the mucus and the PCF, in human tracheobronchial epithelial cell cultures grown in air-liquid interface, were found to travel at the same speed."***

In Methods, information on the data used to inform parameter values from reference 47 has been added (but not for reference 37 for which information was already provided):

> *" The speed of the PCF is believed to be approximately the same as that of the mucus blanket and is estimated to be $v_a \approx 40$ µm/s,* ***based on experiments that measured transport in the PCF and mucus layers, using real-time microscopy to observe fluorescent markers in each layer in vertical profile sections of human tracheobronchial epithelial cell cultures [18]."***

Comment 10. Line 60: the assumption that virus bounces back or is lost at the lower end of the human respiratory tract seems unlikely. Pneumonitis / lower tract disease is one of the feared complications of IAV and in conjunction with bacterial superinfection accounts for most IAV related mortality.

In Methods, we had done a better job explaining this mechanism by stating: *"The bottom boundary condition becomes irrelevant once advection is introduced as the flow at $x = x_{max}$ becomes negligible"*. But our statement in Results on (previous) line 60 was misleading, and while mathematically correct, it is not a good description of the behaviour of our MM. We have replaced it with:

> ***"At the edges of the mathematically modelled HRT, when virus in the PCF reaches the top of the HRT, it is lost (absorptive boundary conditions), and its upwards advection ensures it cannot reach the bottom of the modelled HRT, which becomes irrelevant, as will be shown later."***

Comment 11. Figure 2 & figure 3 d-f: it is impossible to tell the lines apart at higher dpi.

We have added colours to these figures so lines are now clearly distinguishable.

Comment 12. Line 102: how does the new viral production rate (11 * pbaccam) compare to in vitro estimates?

Unfortunately, it is not possible to compare in vivo and in vitro estimates for this parameter. Viral titer measurements in vivo are typically taken from nasal washes and are expressed, for example in Baccam 2006, in units of tissue culture infectious dose 50 ($TCID_{50}$) per mL of nasal wash. To compare this to the virus production rate per cell estimated in vitro, one needs to convert *"per mL of nasal wash"* to *"per infected cell"*. This requires knowing how much liquid bathes all cells in the HRT, how many cells per mL of that liquid are actively infected and virus-producing, what volume was taken out of the patient's respiratory tract as part of the nasal wash vs how much was from the nasal wash liquid itself which was pushed up the patient's nose. Even a ball-park estimate would be misleading rather than helpful, in our opinion.

Also, in our experience, virus production rate estimates, even in vitro, for a given IAV strain, in a given cell culture, performed by the same research team, can vary by > 10-fold between experiments [19].

Comment 13. Lines 115-16: Fig 3g-i makes an interesting prediction. However, I disagree with the strength of the conclusion that depth > 15 cm has little effect as even a 0.5 log increase in peak viral load may be of clinical significance.

It is possible, though we are not aware of any experimental evidence, that a 3-fold increase (0.5 log) in peak viral load has a measurable, clinically significant impact e.g. on a patient's symptoms or on the severity of the immune response it elicits or perhaps something else. However, our position is not that it would have no impact on clinical outcomes, but rather we mean that given the variable/noisy nature of infectious virus concentrations measurements from patient nasal washes, one could not distinguish between two measurements that are only 3-fold different, possibly not even between measurements that are 10-fold different (one-log), especially if one is comparing two different patients. For example, from Baccam 2006, see

TABLE 1. Results of nasal washes taken daily from six volunteers inoculated with $10^{4.2}$ $TCID_{50}$ of cloned wild-type influenza A/Hong Kong/123/77 (H1N1)[a]

Patient	Result for day no.:							
	1	2	3	4	5	6	7	8
1	2.0	5.5	4.0	5.5	3.0	≤0.5	≤0.5	≤0.5
2	1.0	6.0	3.0	1.5	3.5	1.3	≤0.5	≤0.5
3	2.5	5.0	5.0	3.0	5.5	3.5	≤0.5	≤0.5
4	3.5	5.5	6.5	5.5	3.5	4.0	≤0.5	≤0.5
5	2.5	3.0	6.5	6.5	2.0	0.8	≤0.5	≤0.5
6	4.0	5.0	5.5	7.5	5.5	1.3	≤0.5	≤0.5
Avg	2.6	5.0	5.1	4.9	3.8	1.9	≤0.5	≤0.5
SEM	0.4	0.4	0.7	1.7	0.7	0.8	NA	NA

[a] The data are given in $\log_{10}$ $TCID_{50}$/ml of nasal wash. Data are provided by R. Murphy from the study presented in reference 28. NA, not applicable.

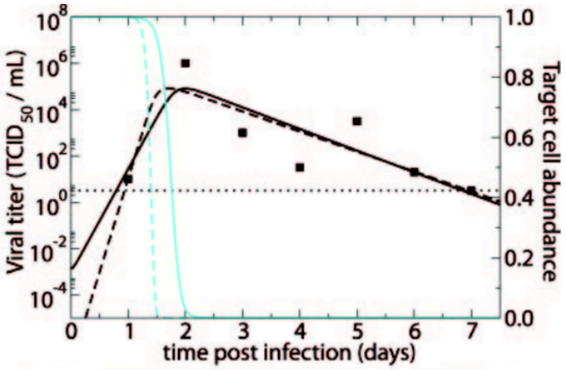

Despite equivalent initial doses received by these volunteers, $\log_{10}(TCID_{50})$ titers varied between 1.0 and 4.0 just one day after inoculation, and peak titers varied between 5.5 and 7.5, a 1000-fold (3-log) and 100-fold (2-log) spread, respectively. While this represents inter-patient variability, and not measurement uncertainty which is harder to estimate, the figure on the right for patient #2 in the same study gives us some idea of uncertainty. If the MM (solid black line) is to be believed, it suggests that titer measurement noise (deviation from the theoretical infection course in this patient) are easily one-log (10-fold) and even perhaps 2-log (100-fold) in magnitude. Having looked at extensive nasal wash time courses from patients, we feel strongly that these errors are certainly at least one-log (e.g. we routinely encounter cases of one sample at 6.0 and the following sample below detection, and the following sample at 5.0).

This being said, it is clear we did not communicate this distinction clearly and have made the following change where we first discuss Fig 3(g–i):

> *" ~~However, the virus titer curve is largely insensitive to the depth of deposition for depths greater than ∼15 cm.~~* ***While such differences in viral titer could have clinical implications, the corresponding changes in viral titer would not be experimentally detectable in light of typical uncertainties (at least 10-fold) in titer measurements from nasal washes."***

and later when we return to this in the Discussion:

> *" While deposition at depths lower than* ∼ 15 cm ***did not result in experimentally measurable differences in*** *the viral titer time course, initial depositions above that point both delayed and reduced peak titer."*

Comment 14. Lines 120-122: I wonder if this conclusion would hold with a stochastic formulation of the model. An interesting feature of a stochastic version of the model may be to predict whether deeper viral site of initial infection is more predictive of successful transmission / acquisition.

With or without stochasticity, advection would act to bring virus from lower locations upwards towards the nose, exposing cells at shallower depths (higher in the HRT) to more virus. Whether, in a stochastic MM, this would result in infections all along that trajectory or only at some random points along the way is unclear, but in particular would depend on the virus production rate of individual cells, the volume of liquid on the airways (dilution volume for the viral progeny), and the susceptibility of cells to infection, some of which would be extremely difficult (maybe impossible) to measure experimentally. The amount of complexity (additional unknown parameters) that a stochastic MM would need resolved in order for its predictions on this point to be believable is quite large. As such, we feel that commenting on whether or not our results would hold or how they would be modulated (amplified or dampened) in a stochastic implementation could be misleading and so we preferred not to.

Comment 15. Lines 134-135: this prediction that infection moves from lower to upper tract despite viral load doing the opposite is a key interesting prediction of the model. However, S2 video only captures this concept somewhat. I suggest creating another video in which the y-axis for fraction of target & infected cells is log-10 converted so that the difference in # of infected cells between the top and bottom of the respiratory tract at early timepoints is more evident.

Yes, indeed, poor accuracy had often been used to mask inconvenient truths! In the interest of accuracy and transparency, we kept S2 Video as is (fractions are easier to think through in linear space) but have added S3 Video where a log-scale is used to show the fraction of target and infectious cells. It shows the same pattern as S2 Video, but provides higher accuracy for the findings. Correspondingly, we changed the text that referenced S2 Video to read:

> *“ This can be seen more clearly in the videos (S2 Video **and S3 Video**) which depict~~s~~ the spatiotemporal course of the infection, in the presence of both diffusion and advection.”*

and added the following as the caption for the S3 Video:

> “ **S3 Video. Same as S2 Video where the fraction of target cells and infectious cells (top row) are shown on a log-scale.** *This video better illustrates that, even when looking at cell concentrations down to one in a million (10^{-6}), the infection still appears to move downwards along the HRT.”*

Comment 16. Figure 4 is very instructive. However, I am left wondering why diffusion is left in the model at all if it is not needed to recapitulate the key kinetic features of observed data.

The reviewer is correct and we could leave diffusion out of the MM after having established this. But one can never be certain it would have a negligible effect under all conditions and circumstances. Given that this is exploratory, developmental work for this MM, we felt it safer to keep diffusion along, for accuracy's sake: it is easier to carry it along than to continuously test that its effect remains negligible. Without having tried it, we wouldn't have known. But this needs to be better communicated in the manuscript so we made the following change:

> *“ The effect of diffusion in the presence of advection is most likely negligible (one part in 10^5 in the fraction of cells infected) in light of typical, in vivo experimental variability (∼10-fold variability). **As such, it could be omitted in future modelling efforts.**”*

Comment 17. Figure 9: these in silico knock out experiments are novel and justify what I would call a rather complex model of immunity relative to most in the field (not simplistic as stated by the authors). However, the authors should mention in the discussion that there are large limitations in terms of extrapolating conclusions from murine models of viral infection to mathematical models intended to capture kinetics of human infection. Lines 264-268 state this well and should be restated as a limitation in the discussion.

We have modified this sentence in the Discussion:

> “ *The spatial MM could also largely reproduce the key features of in vivo immune response knock-out experiments [8]****, although one should be cautious in extrapolating conclusions from murine experiments to humans, and in assuming that experimental disruption of one immune component can be carried out without affecting another.*** ”

Comment 18. Lines 384-386: I may be missing the point, but it seems to me that this underestimate is related to per cell viral production. The model is trained to total measured virus so it is less clear how total viral load is underestimated. Is the idea that the lower tract is not sampled and therefore current clinical studies underestimate total viral load?

The reviewer raises an excellent point! This absolutely needs addressing in our manuscript and we are grateful for the opportunity to rectify this issue.

There are 2 key distinctions to be made here. The first is the difference between the virus titer concentration in the HRT (variable V in our MM) and the virus production rate (parameter p in our MM) which contributes to it. If a cell produces virus at a fixed rate of p virus/hour but virus loses infectivity or is neutralized because it is bound to the mucus, as captured by parameter $c = 0.22\ \mathrm{h}^{-1}$, or is removed via advection (parameter v_a) as it reaches the top of the HRT, then only a fraction of the total virus produced (i.e. what is produced minus what is lost) gets to contribute to the virus concentration ultimately observed in the PCF. So a MM with a higher virus clearance rates (c) or higher advection speed (v_a) would need a higher virus production rate (p) to yield the same observed PCF virus concentration. This is something that was poorly articulated before, but has been addressed in our revised manuscript in response to **Comment 3**: we now show in our manuscript how the virus production rate (p) relates to the advection speed (v_a) to yield equivalent total virus concentration time courses. In the end, this is the reason why, for equivalent total viral load measured, the non-spatial MM underestimates the virus production rate and hence the total virus produced (not total virus measured).

But through their question/answer, the reviewer identifies a second important distinction to be made between the actual virus concentration in the PCF and the virus concentration measured experimentally. To clarify this distinction and explore the impact of this choice, we added the following at the start of Results, after diffusion but before advection:

> “ ***There is an important distinction to be made between the actual virus concentration in the PCF and the virus concentration measured experimentally. In a typical, non-spatial MM, the virus concentration in the PCF is implicitly assumed equal everywhere, $V(t)$, and can be compared directly to the experimentally measured concentration at that time t. In the spatial MM, the virus concentration depends not only on time but also on depth (x), i.e. $V(x,t)$ rather than just $V(t)$. The virus concentration curves shown herein when the x-axis is time rather than depth, correspond to the concentration averaged over all values of $x \in [0, 30]\ \mathrm{cm}$. S1 Figure explores the impact of this choice on our findings below.*** ”

And we return to the implications of this choice for our results in the Discussion:

> “ ***An important distinction between the spatial and non-spatial MMs is that while the latter assumes a spatially uniform virus concentration $V(t)$ that is directly compared against experimental measurements at time t, virus concentration in the present MM depends on both time and depth, $V(x,t)$. Herein, $V(t)$ was obtained by averaging $V(x,t)$ over $x \in [0, 30]\ \mathrm{cm}$ to compare against experiments. This is equivalent to assuming that a patient sample, e.g. a nasal wash, takes a somewhat uniform, mixed up sample of the virus concentration in the HRT down to the $30\ \mathrm{cm}$ represented in the MM. It is likely that typical experimental measurements sample the concentration over a shallower depth. The impact of this choice on our key findings was explored in S1 Figure. Since advection prevents infection from spreading below the deposition depth, decreasing the depth over which $V(x,t)$ is averaged excludes more***

of the infection-free regions where $V(x,t) = 0$. This, in turn, slightly increases $V(t)$, the estimate of the virus concentration that would be measured experimentally. In the presence of the full immune response, averaging only over the top $3\ \mathrm{cm}$ shortened the time for the virus concentration to fall below $1\ \mathrm{TCID_{50}/mL}$ by approximately one day. The spatial MM thus offers a unique opportunity to explore how different HRT sampling methodologies might provide a different picture of the infection course and outcome.”

The corresponding caption for S1 Figure, added as Supplementary Information, is as follows:

> “ **S1 Figure. Effect of varying the length over which the virus concentration is measured.** *The effect of the length, measured from $x = 0$ down to $x = L_{top}$, over which $V(x,t)$ in the spatial MM is averaged to produce the curves showing Virus versus Time, for variants of the MM (a) without cellular regeneration and a full immune response; (b) with cellular regeneration but without a full immune response; and (c) with cellular regeneration and a full immune response.”*

Comment 19. Lines 392-6: as mentioned before, fatal lower tract (lung) disease occurs in a minority of infected persons and is a particular issue in those with T cell immunodeficiency. This argues for some role for diffusion in viral seeding of more distal airways.

In this manuscript, the MM represents the HRT down to a depth of 30 cm, which is somewhere within the bronchi, and so does not include distal airways. Infection kinetics in the very deep regions of the lung falls beyond the spatial extent of our MM. Our “feelings” on this matter is that infection of more distal airways could be the result of the initial deposition depth, rather than a later distal seeding event. Even if experimentally one might first observe an upper HRT infection and only later observe what appears to be spread to the lower HRT, the work herein shows that an infection that starts in the lower HRT and spreads to the upper HRT would still appear as though the distal infection was seeded later, seemingly by the shallower infection. So it is unclear to us whether observation of distal airway infection is the result of later seeding or the late detection of something that was there from the start but had proceeded below detectable levels, and perhaps would have remained so in patients with a non-compromised immune response. But this is merely a conjecture, and since our work focuses on the upper HRT, we would prefer not to speculate on what could be behind experimental observations in the lower HRT. Also, given the physical diffusion rate estimated herein for influenza virions, our MM suggests diffusion cannot overcome the powerful advection, and thus could not cause the distal seeding of the lower HRT, as shows in Fig 4 and Fig 5. So if indeed lower HRT infection are the result of seeding rather than initial deposition, it would have to occur via a mechanism not currently included in our MM.

Comment 20. Lines 406-408, 422-426: These 2 studies showing the trafficking pattern of fluorescent IAV and quantifying % of cells killed during IAV infection are really important for complementing and validating the model's predictions. Until I reached these paragraphs, my impression was that the paper was purely theoretical and hypothesis generating. However, these experiments provide greater weight that the model's central qualitative conclusions are likely to be true. As such, I think it would be fair and useful to mention these papers earlier in the paper, both in the introduction and abstract. I also think it would be great to include a paragraph describing experiments that the authors think would further validate and enhance the model.

We now mention these experimental findings in the Abstract (see our response to **Comment 8**) and in the Introduction, modified as follows:

> *“Via the addition of two spatial parameters whose value is relatively well-established, namely the rate of diffusion and advection of virions, the MM produces a richer range of IAV kinetics, and predicts spatial infection spread patterns consistent with that observed in mice [17, 20]. The further addition of cellular regeneration and*

> ***a simplified immune response allow the MM to reproduce levels of tissue damage consistent with that reported in patients [4]."***

We added the following sentence in the Results section where we first report the finding:

> ***"The observation that the infection appears to be progressing from the top towards the bottom of the HRT has also been noted in experimental studies of mice infected with IAV engineered to be fluorescent or bioluminescent [17, 20]. "***

We tried very hard to think of experiments that could specifically validate the spatial aspects of our MM. Our key hurdle was that we could not think of any reliable experimental method to control or evaluate the deposition depth (where infection is seeded, not where it touches the mucus). One could conduct an experiment in an animal model where a number of animals (for statistical significance) would be sacrificed at very small time intervals, early during the infection, and dissected to stain their respiratory tract for virus to determine the depth at which infection was first seeded, and where it proceeded from there. However, the site where infection is first detectable is likely not the site at which it first began (as we show herein) where infection would likely remain below detection levels. It is also not clear how one would sample the airway surface liquid to measure the virus concentration at various depth, and know how much has been collected and from where (without smearing). This is not obvious even for nasal washes, a point we now make in response to **Comment 18**. Another thought we had is that humans are bipedal whereas animals used to study IAV, mice and ferrets, are quadrapedal, so their respiratory tract is largely horizontal wheras ours is vertical: gravity does not act in the same direction. We are uncertain if or how this would affect the findings or their translatability to humans. Solutions to these problems might exist but we are not sufficiently familiar with the full complement of available experimental techniques to think of ways to overcome them.

On the other hand, we do already (and more so in the revised manuscript) suggest experiments that could challenge the MM further, though not its spatial aspects directly. See the last paragraph of Discussion, where we propose that the addition of a more data-informed immune response would allow the application of the MM to capture/reproduce repeated infections and to explore the localized, antiviral role of IFN. The MM's predictions in these contexts would allow for its further indirect validation, without directly addressing its spatial assumptions.

Comment 21. Given the uncertain early kinetics of H5N1 described by the authors, the modeling of H5N1 seems a bit speculative. Moreover, this section of the paper does not add to the overall premise of the paper, which is that advection is fundamental for understanding the temporo-spatial kinetics of IAV. It seems unlikely that advection would only be important for some but not other strains of IAV.

In our response to **Comment 6**, we address the concerns about this section being speculative, which it is to some extent. But we do not feel this is a problem if it is clearly identified as such, which it is now.

While advection turned out to be the big story of the work presented herein, this is accidental rather than intentional: we did not know it would be when we first set out. Our interest was to showcase what new infection kinetics or results are unlocked when a more spatially accurate MM is considered. We wish to include the section on avian vs human IAV infections because the effect of antiviral therapy (specifically the time window of NAI treatment opportunity in severe infections), is still an important, outstanding question regarding NAIs. As such, the results therein will be of specific interest to pharmaceutical researchers, and provides further MM predictions that can later be challenged by experimental data. The section also very effectively highlights the specific areas where there is a lack of experimental data to inform MMs so as to improve/constrain their predictions.

References

[1] P. Baccam, C. Beauchemin, C. A. Macken, F. G. Hayden, and A. S. Perelson. Kinetics of influenza A virus infection in humans. J. Virol., 80(15):7590–7599, 2 August 2006. doi:10.1128/JVI.01623-05.

[2] C. Beauchemin, S. Forrest, and F. T. Koster. Modeling influenza viral dynamics in tissue. In H. Bersini and J. Carneiro, editors, Proceedings of the 5th International Conference on Artificial Immune Systems (ICARIS 06), number 4163 in Lecture Notes in Computer Science, pages 23–36. Springer-Verlag Berlin Heidelberg, 2006. doi:10.1007/11823940_3.

[3] S. Bermbach, K. Weinhold, T. Roeder, F. Petersen, C. Kugler, T. Goldmann, J. Rupp, and P. König. Mechanisms of cilia-driven transport in the airways in the absence of mucus. Am. J. Respir. Cell Mol. Biol., 51(1):56–67, July 2014. doi:10.1165/rcmb.2012-0530OC.

[4] G. A. Bocharov and A. A. Romanyukha. Mathematical model of antiviral immune response III. Influenza A virus infection. J. Theor. Biol., 167(4):323–360, 21 April 1994. doi:10.1006/jtbi.1994.1074.

[5] X. M. Bustamante-Marin and L. E. Ostrowski. Cilia and mucociliary clearance. Cold Spring Harb. Perspect. Biol., 9(4):a028241, 3 April 2017. doi:10.1101/cshperspect.a028241.

[6] P. Cao, A. W. Yan, J. M. Heffernan, S. Petrie, R. G. Moss, L. A. Carolan, T. A. Guarnaccia, A. Kelso, I. G. Barr, J. McVernon, K. L. Laurie, and J. M. McCaw. Innate immunity and the inter-exposure interval determine the dynamics of secondary influenza virus infection and explain observed viral hierarchies. PLoS Comput. Biol., 11(8):e1004334, 18 August 2015. doi:10.1371/journal.pcbi.1004334.

[7] T. C. Carvalho, J. I. Peters, and R. O. W. III. Influence of particle size on regional lung deposition–what evidence is there? Int. J. Pharm., 406(1-2):1–10, March 2011. doi:10.1016/j.ijpharm.2010.12.040.

[8] H. M. Dobrovolny, M. B. Reddy, M. A. Kamel, C. R. Rayner, and C. A. A. Beauchemin. Assessing mathematical models of influenza infections using features of the immune response. PLoS One, 8(2):e57088, 28 February 2013. doi:10.1371/journal.pone.0057088.

[9] P. Domingo-Calap, E. Segredo-Otero, M. Durán-Moreno, and R. Sanjuán. Social evolution of innate immunity evasion in a virus. Nat. Microbiol., 4(6):1006, 4 March 2019. doi:10.1038/s41564-019-0379-8.

[10] J. V. Fahy and B. F. Dickey. Airway mucus function and dysfunction. N. Engl. J. Med., 363(23):2233–2247, 2 December 2010. doi:10.1056/NEJMra0910061.

[11] A. Handel, I. M. Longini, and R. Antia. Towards a quantitative understanding of the within-host dynamics of influenza A infections. J R Soc Interface., 7(42):35–47, 6 January 2010. doi:10.1098/rsif.2009.0067.

[12] B. P. Holder, L. E. Liao, P. Simon, G. Boivin, and C. A. A. Beauchemin. Design considerations in building in silico equivalents of common experimental influenza virus assays. Autoimmunity, 44(4), June 2011. doi:10.3109/08916934.2011.523267.

[13] Y. Huang, H. Dai, and R. Ke. Principles of robust innate immune response to viral infections: a multiplex network analysis. Front. Immunol., 10:1736, 24 July 2019. doi:10.3389/fimmu.2019.01736.

[14] J. Hussong, R. Lindken, P. Faulhammer, K. Noreikat, K. Sharp, W. Kummer, and J. Westerweel. Cilia-driven particle and fluid transport over mucus-free mice tracheae. J. Biomech., 46(3):593–598, 1 February 2013. doi:10.1016/j.jbiomech.2012.08.020.

[15] A. Iwasaki and P. S. Pillai. Innate immunity to influenza virus infection. Nat. Rev. Immunol., 14(5):315–328, May 2014. doi:10.1038/nri3665.

[16] B. Killingley and J. Nguyen-Van-Tam. Routes of influenza transmission. Influenza Other Respir. Viruses, 7:42–51, September 2013. doi:10.1111/irv.12080.

[17] B. Manicassamy, S. Manicassamy, A. Belicha-Villanueva, G. Pisanelli, B. Pulendran, and A. García-Sastre. Analysis of in vivo dynamics of influenza virus infection in mice using a GFP reporter virus. Proc. Natl. Acad. Sci. U.S.A., 107(25):11531–11536, 22 June 2010. doi:10.1073/pnas.0914994107.

[18] H. Matsui, S. H. Randell, S. W. Peretti, C. W. Davis, and R. C. Boucher. Coordinated clearance of periciliary liquid and mucus from airway surfaces. J. Clin. Invest., 102(6):1125–1131, 15 September 1998. doi:10.1172/JCI2687.

[19] E. G. Paradis, L. T. Pinilla, B. P. Holder, Y. Abed, G. Boivin, and C. A. A. Beauchemin. Impact of the H275Y and I223V mutations in the neuraminidase of the 2009 pandemic influenza virus in vitro and evaluating experimental reproducibility. PLoS One, 10(5):e0126115, 20 May 2015. doi:10.1371/journal.pone.0126115.

[20] V. Tran, L. A. Moser, D. S. Poole, and A. Mehle. Highly sensitive real-time in vivo imaging of an influenza reporter virus reveals dynamics of replication and spread. J. Virol., 87(24):13321–13329, December 2013. doi:10.1128/JVI.02381-13.

[21] A. W. C. Yan, S. G. Zaloumis, J. A. Simpson, and J. M. McCaw. Sequential infection experiments for quantifying innate and adaptive immunity during influenza infection. PLoS Comput. Biol., 15(1):e1006568, 17 January 2019. doi:10.1371/journal.pcbi.1006568.

[22] S. Yang, G. W. Lee, C.-M. Chen, C.-C. Wu, and K.-P. Yu. The size and concentration of droplets generated by coughing in human subjects. J. Aerosol. Med., 20(4):484–494, 2007. doi:10.1089/jam.2007.0610.

[23] Z. Zhang, J. Zhang, K. Huang, K.-S. Li, K.-Y. Yuen, Y. Guan, H. Chen, and W. F. Ng. Systemic infection of avian influenza A virus H5N1 subtype in humans. Hum. Pathol., 40(5):735–739, May 2009. doi:10.1016/j.humpath.2008.08.015.
